# SDH-deficient renal cell carcinoma associated with biallelic mutation in succinate dehydrogenase A: comprehensive genetic profiling and its relation to therapy response

**DOI:** 10.1038/s41698-018-0053-2

**Published:** 2018-03-20

**Authors:** Christopher R. McEvoy, Lisa Koe, David Y. Choong, Huei San Leong, Huiling Xu, Deme Karikios, Jeffrey D. Plew, Owen W. Prall, Andrew P. Fellowes, Stephen B. Fox

**Affiliations:** 1 Department of Pathology, Peter MacCallum Cancer Centre, Melbourne, Victoria, 3000 Australia; 2 Clinical Biochemistry and Molecular Genetics, NSW Health Pathology, St Leonards, NSW 2065 Australia; 3 Clinical Pathology, Faculty of Medicine, Dentistry and Health Science, The University of Melbourne, Parkville, VIC 3010 Australia; 4 Nepean Cancer Care Centre, Sydney, NSW 2747 Australia; 5 Department of Radiology, Nepean Hospital, Sydney, NSW 2747 Australia

## Abstract

Succinate dehydrogenase (SDH)-deficient renal cell carcinoma (RCC) is a rare RCC subtype that is caused by biallelic mutation of one of the four subunits of the SDH complex (*SDHA*, *B*, *C*, and *D*) and results in inactivation of the SDH enzyme. Here we describe a case of genetically characterized SDH-deficient RCC caused by biallelic (germline plus somatic) *SDHA* mutations. *SDHA* pathogenic variants were detected using comprehensive genomic profiling and SDH absence was subsequently confirmed by immunohistochemistry. Very little is known regarding the genomic context of SDH-deficient RCC. Interestingly we found genomic amplifications commonly observed in RCC but there was an absence of additional variants in common cancer driver genes. Prior to genetic testing a PD-1 inhibitor treatment was administered. However, following the genetic results a succession of tyrosine kinase inhibitors were administered as targeted treatment options and we highlight how the genetic results provide a rationale for their effectiveness. We also describe how the genetic results benefited the patient by empowering him to adopt dietary and lifestyle changes in accordance with knowledge of the mechanisms of SDH-related tumorigenesis.

## Introduction

Succinate dehydrogenase (SDH) is a key respiratory enzyme complex that converts succinate to fumarate in the citric acid cycle (CAC) and also functions in the mitochondrial electron transport chain. It comprises 4 subunits, SDHA, SDHB, SDHC, and SDHD, which are each transcribed by separate nuclear genes. Cellular SDH deficiency is associated with a distinct array of tumor types, including pheochromocytoma/paragangliomas, gastrointestinal stromal tumors, and (more rarely) renal cell carcinomas (RCCs). The mechanism of SDH-deficient tumorigenesis appears to involve the accumulation of succinate in the cytosol and its subsequent oncogenic effects caused by both hypoxia inducible factor (HIF)-α prolyl hydroxylase inhibition^1^ and the induction of genome-wide hypermethylation due to TET enzyme inhibition.^2,3^

SDH-deficient RCCs were first recognized as a provisional entity by the 2013 International Society of Urological Pathology (ISUP) Vancouver Classification.^4^ They are rare, with an estimated frequency of 0.05–0.2% amongst all RCCs, and they display distinct clinical, morphologic, and molecular features.^5^ Furthermore, within this rare RCC group SDH deficiency due to biallelic *SDHB* loss appears to be most frequent while biallelic *SDHA* loss has rarely been reported.^5,6^ Little is known regarding the genomic context of SDH-deficient RCC and how it relates to therapeutic options. Here we describe a case of SDH-deficient RCC caused by biallelic (germline plus somatic) functional loss of *SDHA*. We also describe how the results of comprehensive genetic testing provided a rationale for the effectiveness of various treatment decisions and the positive impact this had on the patient.

## Case report

Following investigation for hematuria, a 45-year-old male was diagnosed with what was originally considered stage III type 2 papillary RCC. The patient was treated with laparoscopic nephrectomy with curative intent. No evidence of metastatic disease was detected on CT scans prior to, or 3 months after, surgery. Recurrent disease was first detected on a PET-CT scan 10 months after surgery, with evidence of peritoneal, retroperitoneal, subcutaneous, and pulmonary metastases. The patient reported no significant family history of cancer.

**Fig. 1:**
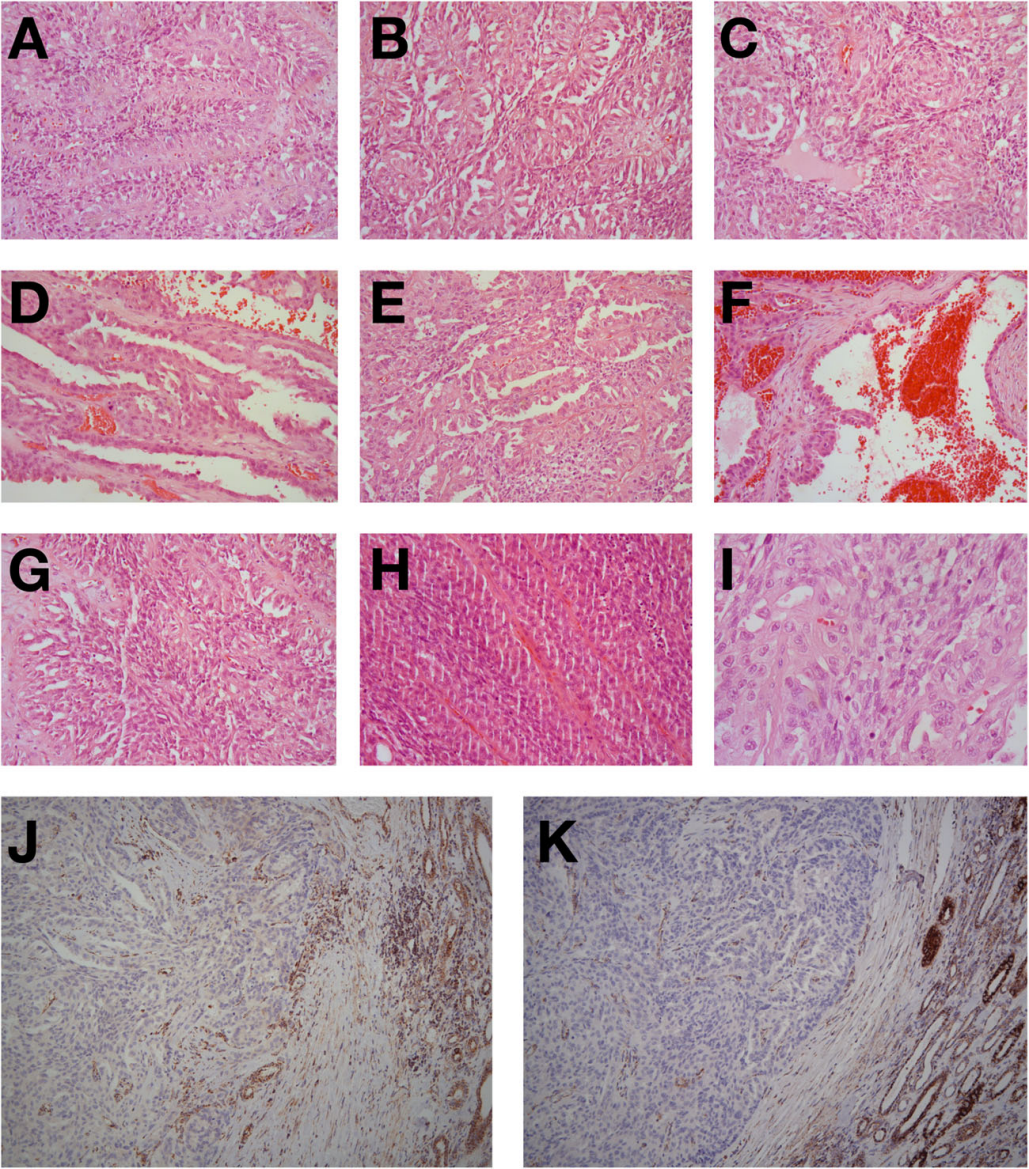
Tumor morphology and IHC results as discussed in the text. Pseudo-papillae and perivascular pseudo-rosettes (**a–c**), papillae (**d**), solid foci and papillae (**e**), microcysts (**f**), spindle cell foci and pseudo-papillae (**g**), pure spindle cell areas (**h**), eosinophilic cytoplasmic inclusions (arrows in **i**). Immunohistochemical analysis demonstrates negative staining for SDHA (**j**) and SDHB (**k**). Internal controls show SDHA and SDHB staining in nonneoplastic tissue

Gross examination revealed an 11 cm solid tan and focally hemorrhagic tumor invading renal parenchyma, the renal vein and sinus fat. H&E sections from the nephrectomy showed that the tumor was encapsulated and did not incorporate benign renal tubules. There was patchy coagulative necrosis. The tumor showed a variety of morphologies that were present in most blocks. It predominantly formed perivascular pseudo-papillae and pseudo-rosettes, with some solid sheets and true papillae, separated by small amounts of hyalinized stroma. There were occasional microcysts at the tumor periphery. Tubules were rarely seen. Tumor cells had dense eosinophilic, somewhat flocculent, cytoplasm and pleomorphic irregular nuclei with coarse chromatin and prominent nucleoli. Narrow fascicles of spindle cells were present throughout the tumor, representing sarcomatoid dedifferentiation, ISUP grade 4. These were mostly located between, and in direct contact with, the epithelial components, with one area of expansile pure spindle cell growth. Very rare clusters of cytoplasmic inclusions were only identified following a careful search in 17 blocks of tumor. Examples of the described morphologies are depicted in Fig. 1 a–i.

On immunohistochemical evaluation the tumor cells showed strong and diffuse expression of AE1/AE3, CAM5.2, EMA, PAX8, CD10, and AMACR. Tumor cells showed very weak staining with an antibody for RCC Ma (renal cell carcinoma marker), and CK20 stained only very rare cells. They were negative for CK7, vimentin and KIT. Only very rare KIT + intratumoral mast cells were seen, fewer than in surrounding benign parenchyma. SDH loss is routinely detected by immunohistochemistry against SDHB since the loss of any SDH subunit results in instability of the entire complex and degradation of SDHB^7,8^ Immunohistochemical staining of an SDHA-deficient tumor therefore reveals an absence of both SDHA and SDHB signal. This was confirmed (Fig. 1j, k) and the diagnosis was amended to SDH-deficient RCC.

Genetic testing comprised the analysis of 2.34 Mb genomic regions implicated in cancer, including sequencing of the entire coding regions of 386 genes. Copy number changes were also analyzed. Genes specifically implicated in renal neoplasia, including *VHL*, *BAP1*, *NF2*, *NFE2L2*, *PBRM*, *MTOR*, *SETD2*, *TSC1*, *FH*, *FLCN*, *MET*, *PIK3CA*, *PTEN*, and all *SDH* subunits were included, as were genomic regions informative for common gene fusions, microsatellite instability, drug efficacy and toxicity, and UV damage. Both tumor DNA extracted from FFPE tissue sections and germline DNA extracted from peripheral blood were analyzed. KAPA Hyper libraries were prepared and target enriched using SureSelect^XT^ hybridization. Pooled library pairs were sequenced at 500 × /100 × mean coverage (tumor/blood) on an Illumina NextSeq sequencer using paired 75 bp reads.

Our genetic analysis identified two variants in *SDHA* (Refseq accession number SDHA NM_004168.2). These consisted of a germline truncating variant c.91 C > T (p.Arg31*), in conjunction with a somatic missense variant c.1765C > T (p.Arg589Trp). Both of these variants are predicted to seriously compromise SDH function. The germline variant produces a truncation of the protein while the somatic variant has been classified as “likely pathogenic” in a recent in silico analysis.^9^ No variants in other common oncogenes or tumor suppressor genes were detected. Somatic copy number analysis detected chromosomal gains of 2p (3 copies), 7p (4 copies, including *EGFR*), 12p (3 copies, including *KRAS*), and 17 (3 copies, including *MAP2K4*), and monosomy of 9, 13q, and 15q. No gene fusions or mutational signatures of clinical significance were detected.

**Fig. 2:**
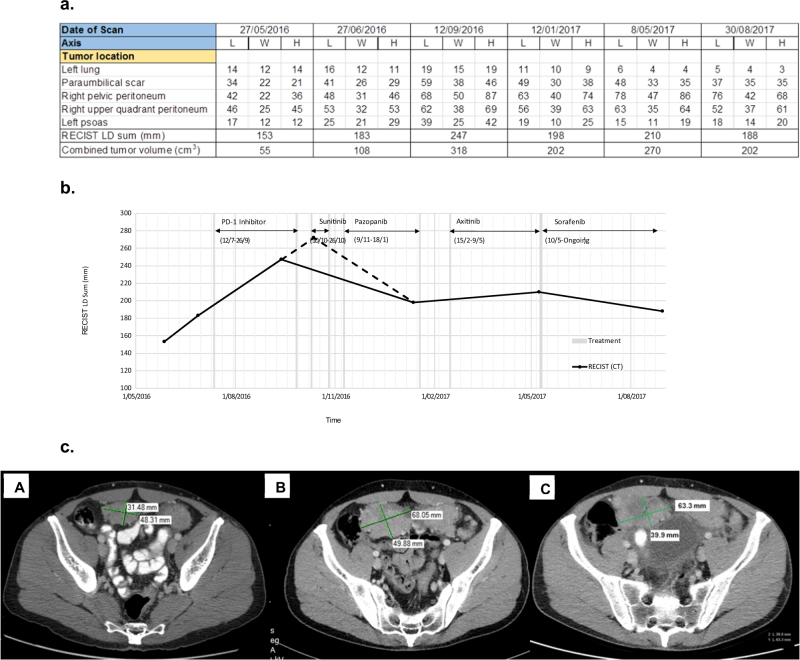
Tumor response to therapeutic treatments. Prior to treatment with PD-1 inhibitor, chest and abdominal PET-CT scans demonstrated peritoneal, retroperitoneal, subcutaneous, and pulmonary metastases. **a** Table of the longest axial diameter (LD) measurements (mm) of length (L), width (W), height (H), based on RECIST^27^ and calculated volume estimates of 5 target lesions taken during the course of the disease. **b** Time course of CT evaluation of tumor response (RECIST scores from Fig. 2a) with relation to various treatments. An extrapolated point was obtained by projecting initial tumor growth to the date of commencement of the first TKI therapy (sunitinib). The resulting portion of the graph is indicated with broken lines. **c** CT image examples of a target right pelvic peritoneum lesion. **A** 27 June 2016, prior to commencement of PD-1 inhibitor trial. **A** 12 September 2016, during PD-1 inhibitor trial. **C** 12 January 2017, following sunitinib and pazopanib treatment

Prior to genetic testing the patient had participated in a clinical trial of a PD-1 inhibitor (ClinicalTrials.gov Identifier: NCT02407990) but the tumor was unresponsive over a 10 week period. Following genetic testing sunitinib, a tyrosine kinase inhibitor (TKI), was administered. A partial clinical response was observed but severe skin rash necessitated drug withdrawal after 16 days and the multikinase angiogenesis inhibitor pazopanib was subsequently given. Again a partial clinical response was observed but acute drug hepatotoxicity necessitated intermittent treatment which was eventually withdrawn after 10 weeks. Axitinib was then administered for 3 months with limited response. A fourth TKI, sorafenib, which has been used for 7 months at the time of writing, has maintained stable disease with only modest side effects. Details of the tumor response to these treatments are shown in Fig. 2. It reveals tumor growth during PD-1 treatment followed by stabilization following treatment with sunitinib, pazopanib, and sorafenib.

At the time of commencing sunitinib treatment the patient also self-initiated a low carbohydrate diet based on an understanding of SDH function and succinate accumulation as the basis for increased HIFs. Interestingly, the patient reported significant subjective improvement in clinical fatigue.

The patient consented to all tests and treatments described in this study and written informed consent was obtained from the patient regarding the publication of his case details.

## Discussion

Here we describe the detection, analysis (genetic, histologic, and immunohistochemical), treatment, and treatment monitoring, of an SDHA-deficient RCC following a 2 year period from initial detection and surgery. SDH-deficient RCC due to SDHA deficiency has rarely been documented. Indeed, two recent studies using large SDH-deficient RCC case series have emphasized that within this rare disease subtype, SDHB deficiency predominates while SDHA deficiency is extremely infrequent. Williamson and colleagues investigated 37 tumors with features morphologically suggestive of SDH-deficient RCC.^6^ Immunohistochemical analysis indicated that 11 of these tumors had lost SDHB expression while only one of the 11 revealed SDHA loss. The genetic basis for SDHA loss was not determined. In the second study immunohistochemistry was used to identify 36 SDHB-deficient RCCs from 27 patients.^5^ All 36 were positive for SDHA expression and germline *SDHA*, *B*, *C*, and *D* mutation testing of 17 patients found 16 *SDHB* mutations, 1 *SDHC* mutation and no mutations in either *SDHA* or *D*. This study also included a summary of 53 previously reported RCC patients exhibiting an *SDH* mutation. Of these, 41 cases involved an *SDHB* mutation, 5 involved an *SDHC* mutation, 3 involved an *SDHD* mutation, and 0 involved an *SDHA* mutation.^5^

SDH typically functions as a classical “two-hit” tumor suppressor where an inactivating germline mutation in one allele is associated with the acquired somatic inactivation of the remaining allele. To our knowledge this report represents the first description of this process involving *SDHA* in RCC. However, two recent cases have concluded that SDHA can also be inactivated in a purely somatic manner. The first involved a case of SDH-deficient RCC that was found to possess a somatic homozygous deletion of 9 *SDHA* exons,^10^ while the second involved a somatic single nucleotide splice site alteration.^11^ It is intriguing that neither of these reports describe the classic paradigm of a biallelic germline plus somatic mutation that we describe here, and that follows for all previously reported SDH-deficient RCCs.^5^ It is currently unclear whether this represents a true SDHA-specific anomaly or is simply the result of our limited current understanding of the genetic basis for SDHA loss.

Little is known regarding the genomic context of SDH-deficient RCC. However, the genetic characterization of papillary RCC (both type I and II) has shown that copy number changes play a significant role in tumorigenesis.^12,13^ Specifically, copy number gains on chromosomes 7 and 17q are common.^12–14^ In keeping with this finding, we detected tetraploidy of 7p (which contains the oncogene *EGFR*) and triploidy of chromosome 17. Our results support the hypothesis that RCC is associated with a limited number of variants in common cancer driver genes and that copy number variations play an important role.

The tumor showed mixed solid, papillary and microcystic architecture, and flocculent eosinophilic cytoplasm. These histological features are evident in most reported high grade SDH-deficient tumors, although these have been predominantly SDHB-deficient, with only small numbers of SDHA- or SDHC-deficient high grade RCCs reported.^5,10,11,15^ This suggests that their identification, particularly in tumors with eosinophilic cytoplasm and that are CK7-negative and KIT-negative by immunohistochemistry, should serve as a useful prompt to a pathologist to initiate immunohistochemistry screening for SDH deficiency. However, the future identification of larger numbers of high grade SDH-deficient RCCs by un-biased methods will help to determine whether these features are typical. Features characteristic of ISUP grade 2 SDH-deficient tumors, including nested growth, uniform cytology, and neuroendocrine-like chromatin,^5,6^ were all absent, consistent with the absence of low grade foci in the current tumor. Intracytoplasmic vacuoles and inclusions appear to be most prominent in low grade SDH-deficient tumors^5^ and consistent with this they were only identified following extensive sampling and a dedicated search in the current tumor. Prominent numbers of intratumoral mast cells have been reported in all grades of SDH-deficient RCC,^5,10,11,15^ but were not seen in the current case. Therefore, whilst the presence of cytoplasmic inclusions and mast cells is very useful, their absence, particularly in high grade or poorly sampled RCC, should not deter pathologists from screening for SDH-deficiency.

There are currently no treatments available for papillary RCC on the Australian government funded pharmaceutical benefits scheme. The patient was therefore initially referred to a clinical trial for a PD-1 inhibitor (nivolumab) as there is evidence for its effectiveness in papillary RCC.^16^ The effectiveness of PD-1 inhibitors correlates well with tumor mutational burden and the finding of a modest overall number of mutations in this case provides a possible explanation for its ineffectiveness. Following genetic testing the patient was treated with a succession of TKIs starting with sunitinib. Several clinical trials have demonstrated the effectiveness of sunitinib against metastatic RCCs,^17–19^ and its effectiveness in cases of SDH-deficient metastatic papillary RCC and SDH-deficient metastatic RCC, type unclassifiable, have also been reported.^20,21^ This is not surprising since tumorigenesis caused by SDH deficiency is achieved via a pseudohypoxic pathway involving HIFs, of which VEGF is a target. Each of the four TKIs administered here are inhibitors of the VEGF receptor and are FDA approved for use in RCC.^19,22–24^ Furthermore, TKIs are also standard therapy in cases of EGFR overexpression. Thus, the genetic results provide a rationale of why TKIs were a more appropriate treatment choice in this case.

Genetic profiling identified a germline mutation associated with familial cancer risk that enabled first degree relatives to receive genetic counselling. The genetic information was also perceived as beneficial by the patient as it increased his knowledge and understanding of his condition. The patient was keen to actively participate in his clinical management through diet and lifestyle modifications and has reported significant subjective improvement in clinical fatigue following the administration of a low carbohydrate diet. This anecdote should be treated with circumspection since the diet was commenced simultaneously with sunitinib treatment. However, there has been increased clinical interest on the potential of targeting tumors through their increased reliance on glycolysis metabolism. Whilst there is debate whether the Warburg effect applies generally to all tumors, it has been proposed that tumors with inactivating mutations in genes encoding CAC enzymes, including *SDH* and fumarate hydratase, are forced to use glycolysis as the major source of energy production, due to incapacitation of CAC energy production.^25^ The accumulation of succinate in SDH deficiency inhibits HIF prolyl hydroxylase, leading to HIF accumulation even under normoxic conditions.^1^ Increased intracellular HIF, particularly HIF-1α, leads to transcriptional upregulation of a variety of genes including *GLUT1*, *PFK2*, *PDH*, and *LDH-A*, which promote aerobic glycolysis.^25,26^ These findings suggest that a low carbohydrate diet, as adjuvant therapy for SDH deficient tumors, may warrant further investigation.

In summary, we provide a detailed description of the extremely rare entity of SDHA-deficient RCC, with biallelic (germline plus somatic) *SDHA* mutations as a unique feature. Comprehensive genetic testing elucidated the underlying pathogenesis of RCC in this patient and demonstrated limited variation in common cancer driver genes but high gene copy number variation. We also show how the genetic results correlated well with clinical responses to therapeutic agents and provided a rationale for their effectiveness. Furthermore, we demonstrate the positive effect that genetic testing can have on the patient by allowing their active participation in clinical management, and also highlight its impact on the patient’s relatives by allowing them to undergo genetic counselling and preventative medicine.

### Data availability

The data that support the findings of this study are available on request from the corresponding author (CRM). The data are not publicly available due to privacy restrictions as they are part of a private medical record.
